# A case report depicting patient’s installation on the fracture table when an ankle spanning external fixator is already in place

**DOI:** 10.1186/s12891-019-2808-5

**Published:** 2019-09-04

**Authors:** Morad Mohamad, Alexandre Ansorge, Diogo Vieira Cardoso, Axel Gamulin

**Affiliations:** Division of Orthopaedic and Trauma Surgery, Department of Surgery, University Hospitals of Geneva, 4 Rue Gabrielle-Perret-Gentil, CH-1211, Geneva 14, Switzerland

**Keywords:** Fracture table, Traction table, Ankle spanning external fixator, Fracture reduction and fixation, Total hip arthroplasty

## Abstract

**Background:**

Fractures of the proximal and diaphyseal femur are frequently internally fixed using a fracture table. Moreover, some femoral neck fractures may be treated with total hip arthroplasty using a direct anterior approach and a traction table. Fracture and traction tables both use a boot tightly fitted to the patient’s foot in order to: 1) obtain fracture reduction by traction and adequate rotation exerted on the slightly abducted or adducted extremity; or 2) adequately expose the hip joint using traction, rotation and extension to implant total hip arthroplasty components. In some instances, multiply injured patients may present with both a proximal or diaphyseal femur fracture and a diaphyseal or distal tibia or ankle fracture necessitating an ankle spanning external fixator on the same limb. Frequently, the tibia or ankle fracture has to be treated first, and standard use of the fracture or traction table may be thereafter difficult due to the external fixator construct preventing tight fitting of the boot to the patient’s foot.

**Case presentation:**

In order to address this situation, the authors describe a simple technique allowing rigid fixation of the limb with an ankle spanning external fixator to the traction or fracture table, providing accurate control of the position of the lower limb in all planes for adequate fracture reduction and fixation or total hip arthroplasty. The technique is exemplified with a clinical case.

**Conclusions:**

This technique allows an efficient way to: 1) timely stabilize diaphyseal or distal tibia or ankle fractures; and 2) subsequently use all the advantages of a fracture or traction table to adequately reduce and fix proximal or diaphyseal femur fractures, or optimally expose femoral neck fractures for total hip arthroplasty using a direct anterior approach.

## Background

**Fig. 1:**
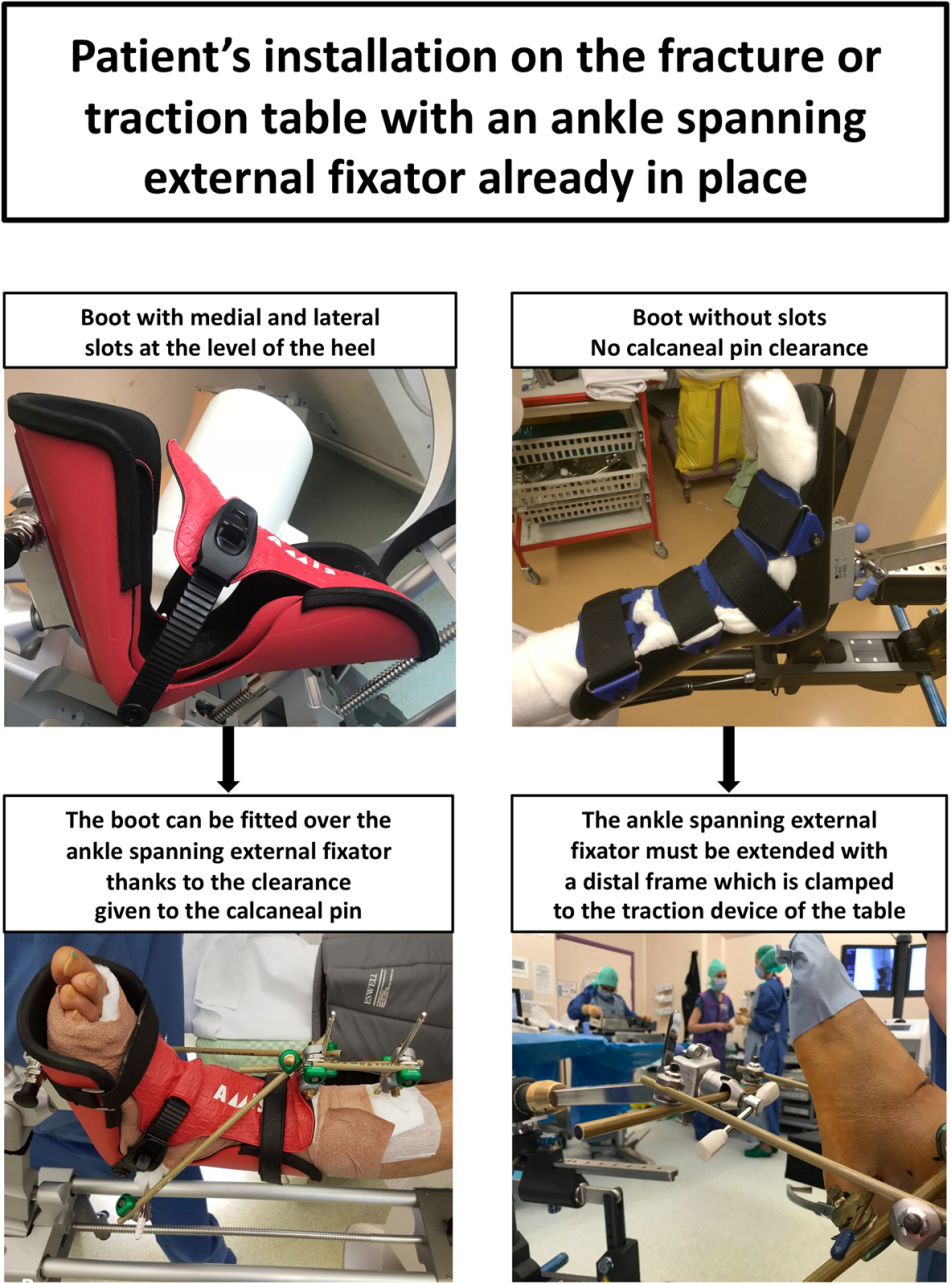
Modified version of the poster used in the authors’ institution as a technical reminder. This version was translated into English from the original version, and institutional markings were removed. On the left side, example of the AMIS Mobile Leg Positioner boot (Medacta International SA, Castel San Pietro, Switzerland): the boot has medial and lateral slots allowing proper fitting of the patient’s distal leg into the boot, despite the presence of a tibio-cacaneal external fixator with a transcalcaneal pin. On the right side, example of the boot used with the operating table TruSystem™ 7500 with extension unit (Trumpf Medical, Saalfeld, Germany): the boot has no medial or lateral slot and the external fixator construct must be extended with a distal frame (Hoffmann III, Stryker, Selzach, Switzerland) which can be inserted and clamped into the fixation apparatus dedicated to the attachment of the boot

Fractures of the proximal and diaphyseal femur are most often closely reduced and internally fixed with the well-established use of a fracture table [1–6]. Stable fracture reduction is obtained with axial traction, adequate rotation and slight abduction or adduction exerted on the extremity prior to internal fixation, with the occasional aid of an additional percutaneous or limited open approach to achieve optimal bone fragment positioning [1–6]. Moreover, some femoral neck fractures may be treated with hemiarthroplasty or total hip arthroplasty (THA), depending on the fracture displacement and/or comminution and the physiologic status of the patient [7–13]. In some institutions, THA are routinely implanted using a direct anterior approach and a traction table adequately exposing the hip joint using traction, rotation and extension [14–17]. Both fracture and traction tables usually use a boot tightly fitted to the patient’s foot and rigidly connected to the traction device of the table (Fig. 1). This provides an accurate and stable control of the amount of traction, internal or external rotation, abduction or adduction, and extension or flexion of the lower extremity.

In some instances, multiply injured patients may present with both a proximal or diaphyseal femur fracture (treated either with a closed reduction and internal fixation on a fracture table or with a THA using a traction table) and a diaphyseal or distal tibia or ankle fracture necessitating an ankle spanning tibio-calcaneal or tibio-talar external fixator on the same limb [18–24]. Frequently, the tibia or ankle fracture has to be treated first, especially when open, dislocated or associated with acute compartment syndrome [18–24]. Standard use of the fracture or traction table may be thereafter difficult due to the external fixator construct preventing proper fitting of the boot to the patient’s foot, especially if the boot has no medial and lateral slots giving clearance to the transcalcaneal pin (Fig. 1).

In order to address this situation, the authors describe a simple technique allowing rigid fixation of the limb with an ankle spanning external fixator to the traction device of the fracture or traction table, providing accurate control of the position of the lower limb in all planes for adequate fracture fixation or THA implantation. The technique is exemplified with a clinical case.

## Case presentation

A 52 year-old male patient was admitted after a high-velocity road traffic accident where he sustained among other injuries a left open Gustilo and Anderson grade II, segmental femoral shaft fracture type AO/OTA 32C2(i), a left open Gustilo and Anderson grade II, comminuted patella fracture type AO/OTA 34C2, a left closed, proximal intra-articular tibia fracture type AO/OTA 41B1.1 associated with a proximal fibula fracture type AO/OTA 4F1A(n), and a left closed, distal extra-articular tibia fracture type AO/OTA 43A3.3 associated with a distal fibula fracture type AO/OTA 4F3B [25, 26].

Wounds were débrided and irrigated, a left femoro-tibio-calcaneal external fixator was applied (Hoffmann III, Stryker, Selzach, Switzerland), and the patella fracture was fixed using the tension band wire technique on day 0, following damage control orthopedics protocol [27].

**Fig. 2:**
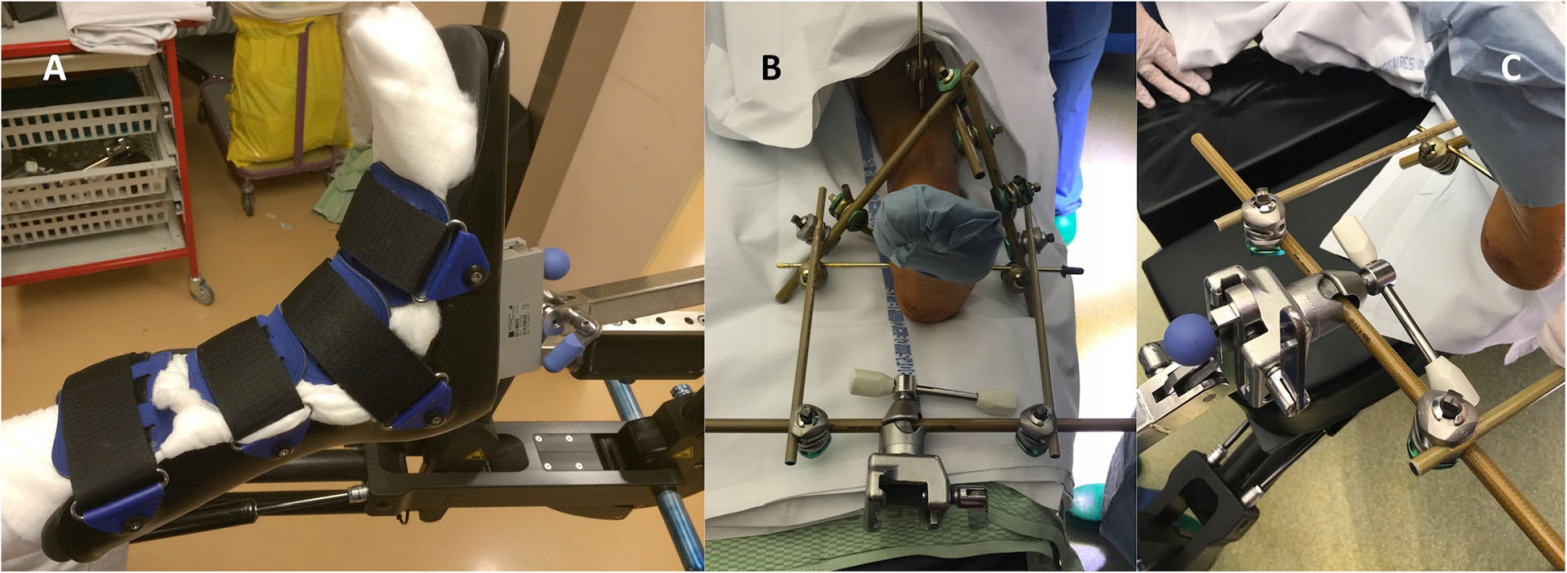
**a** the boot used in this example (operating table TruSystem™ 7500 with extension unit, Trumpf Medical, Saalfeld, Germany) has no medial and lateral slot giving clearance to a pin inserted into the middle part of the posterior tuberosity of the calcaneus. **b** the external fixator construct is extended with a distal frame consisting of three rods and four coupling devices (Hoffmann III, Stryker, Selzach, Switzerland). **c** the extended frame is fixed to the traction device of the fracture table by inserting and clamping connecting rods into the fixation apparatus that is usually dedicated to the attachment of the boot

Definitive treatment of the femoral shaft fracture and proximal tibia fracture was delayed until day 7, but local soft tissue conditions around the distal tibia did not allow formal open reduction and internal fixation due to unhealed blisters. For this reason, the tibio-calcaneal external fixator could not be removed. After general endotracheal anesthesia and intravenous administration of Cefuroxime, the patient was positioned supine on a radiolucent table. The patient was prepared and draped for percutaneous screw fixation of the left proximal tibia fracture. The patient was then re-positioned on the fracture table for anterograde femur intramedullary nailing. The femoral part of the remaining external fixator was removed, leaving only the tibio-calcaneal construct. The positioning of the transcalcaneal pin did not allow proper fitting of the fracture table boot to the foot of the patient: the pin was inserted into the middle part of the posterior tuberosity of the calcaneus and the boot had no medial and lateral slot giving clearance to the pin (Fig. 2a: operating table TruSystem™ 7500 with extension unit, Trumpf Medical, Saalfeld, Germany). For this reason, the external fixator construct was extended with a distal frame consisting of three rods and four coupling devices (Hoffmann III, Stryker, Selzach, Switzerland) (Fig. 2b). Subsequently, the extended frame was fixed to the traction device of the fracture table by inserting and clamping connecting rods into the fixation apparatus that is usually dedicated to the attachment of the boot (Fig. 2c). Traction, rotation and slight abduction were then applied under fluoroscopic control in order to obtain adequate and stable reduction of the fracture. Surgery was then carried out after proper preparation and draping. An anterograde T2-recon femoral nail was inserted following the usual technique (Stryker, Selzach, Switzerland). After fluoroscopic control of the fracture reduction and fixation, copious irrigation was performed and the wounds were closed. Staples were used to close the skin. Sterile dressing was applied. No drains were used. The external fixator construct was disconnected from the fracture table traction device and the dedicated frame extension was dismantled. Upon operating room discharge, the lower extremity was well perfused and distal pulses were present. Postoperative radiographs showed adequate fracture reduction and fixation, as well as adequate femoral rotation.

Definitive treatment of the distal tibia fracture occurred in another institution, after the patient was transferred due to international health insurance regulations.

## Discussion and conclusions

Multiply injured patients with both a proximal or diaphyseal femur fracture and a diaphyseal or distal tibia or ankle fracture on the same limb might represent management and positioning issues in the operating room. The distal limb skeleton must be adequately stabilized to use the fracture or traction table in order to obtain proper fracture reduction for proximal femoral fixation or optimal surgical exposition for THA implantation. In some instances, definitive tibia or ankle fracture fixation using intra-medullary nailing or plate and screws may be contra-indicated because of soft-tissue conditions (local damage control) [18–24] or increased time consumption (general damage control) [27], and ankle spanning external fixation has to be performed as the first surgical step.

The most widely used method for applying ankle spanning external fixation consists of a tibio-calcaneal construct (delta or Y frame), often extended with talar or metatarsal pins [28]. Using this technique, the calcaneal pin is inserted in the most solid part of the posterior tuberosity of the calcaneus, in order to achieve good bone purchase, avoid medial neurovascular structures injury, and act against equinus deformity of the ankle [28, 29]. Most of the boots fitted to usual fracture and traction tables do not have slots deep enough at the level of the heel to accommodate a posterior and plantar calcaneal pin, except for some specific models (Fig. 1). For this reason, the usual external fixator construct should be extended with a distal frame that can be fixed into a clamp mounted on the traction device of the table, using a technique similar to the one previously published for fixation of an amputated lower limb to a fracture table (Fig. 2) [30]. As this construct can rely on atypical adaptation of rarely used fracture or traction table accessories, the authors recommend a blank trial with the in-house usual fracture and traction tables before the first surgery is undertaken.

Alternatively, ankle spanning external fixation can be performed using a talar neck pin, for example when the calcaneus is fractured and cannot be used for pin insertion [28, 31]. The more anterior and dorsal positioning of the talar pin, when compared to a calcaneal pin, might permit easier fitting of the traction device boot to the foot in some instances, allowing the patient to be installed on the fracture or traction table in the usual way. If this is not possible, the external fixator construct should be extended as previously described in this paper (Fig. 2). The authors do not recommend recognizing the potential advantage of talar pin placement in terms of easier fracture or traction table boot fitting as an isolated indication to use this technique rather than the usual calcaneal pin insertion method.

In the above described case, two important concepts in the treatment of trauma patients were met. First, damage control orthopedics concepts were applied when dictated by the general condition of the patient or the local soft tissue condition. External fixation was used as the first management step in order to obtain optimal outcome. Secondly, despite an external fixation construct hindering usual use of a fracture or traction table, accurate and stable control of the position of the lower limb in all planes for adequate fracture fixation or THA implantation and optimal postoperative outcome could be obtained.

In conclusion, the authors present a simple technique to use the fracture or traction table with an ankle spanning external fixator already in place (Figs. 1 and 2). This allows an efficient way to: 1) timely stabilize diaphyseal or distal tibia or ankle fractures especially when open, dislocated or associated with an acute compartment syndrome; and 2) subsequently use all the advantages of a fracture or traction table to adequately reduce and fix proximal or diaphyseal femur fractures, or optimally expose femoral neck fractures for THA implantation using a direct anterior approach. The technique presented in this paper does not rely on any specific model of external fixator or boot used with fracture or traction table, and can be widely used in different operative room settings. The authors recommend a blank trial with the in-house usual fracture and traction tables and accessories before the first surgery is undertaken.

## Data Availability

The datasets used and/or analysed during the current study are available from the corresponding author on reasonable request.

## Abbreviations

AO: Arbeitsgemeinschaft für Osteosynthesefragen
OTA: Orthopaedic Trauma Association
THA: total hip arthroplasty
